# Cystathionine beta-synthase null homocystinuric mice fail to exhibit altered hemostasis or lowering of plasma homocysteine in response to betaine treatment

**DOI:** 10.1016/j.ymgme.2010.06.007

**Published:** 2010-10

**Authors:** Kenneth N. Maclean, Jakub Sikora, Viktor Kožich, Hua Jiang, Lori S. Greiner, Eva Kraus, Jakub Krijt, Linda S. Crnic, Robert H. Allen, Sally P. Stabler, Milan Elleder, Jan P. Kraus

**Affiliations:** a Department of Pediatrics, University of Colorado School of Medicine, Aurora, CO, United States; b Institute of Inherited Metabolic Diseases, Charles University, 1st Faculty of Medicine, Prague, Czech Republic; c Department of Medicine, University of Colorado School of Medicine, Aurora, CO, United States

**Keywords:** ALT, Alanine aminotransferase, aPTT, activated partial thromboplastin time, BHMT, betaine-homocysteine *S*-methyltransferase, HCU, classical homocystinuria, CBS, cystathionine beta-synthase, CGL, cystathionine gamma-lyase, DMG, dimethylglycine, ER, endoplasmic reticulum, fHcy, free homocysteine, Hcy, homocysteine, LDH, lactate dehydrogenase, MG, methylglycine, NAC, *N*-acetylcysteine, PT, prothrombin time, AdoMet, *S*-adenosylmethionine, AdoHcy, *S*-adenosylhomocysteine, tHcy, total homocysteine, Betaine, Coagulation, Cystathionine, Cystathionine beta-synthase, Cystathionine gamma-lyase, Homocystinuria, Homocysteine

## Abstract

Cystathionine beta-synthase (CBS) deficient homocystinuria is an inherited metabolic defect that if untreated typically results in mental retardation, thromboembolism and a range of connective tissue disturbances. A knockout mouse model has previously been used to investigate pathogenic mechanisms in classical homocystinuria (Watanabe et al., PNAS 92 (1995) 1585–1589). This mouse model exhibits a semi-lethal phenotype and the majority of mice do not survive the early neonatal period. We report here that the birth incidence of *cbs* (−/−) mice produced from heterozygous crosses is non-Mendelian and not significantly improved by treatment with either the Hcy lowering compound betaine or the cysteine donor *N*-acetylcysteine. Betaine treatment did improve survival of cbs (−/−) mice and restored fertility to female *cbs* (−/−) mice but did so without significantly lowering Hcy levels. Surviving *cbs* (−/−) mice failed to show any alteration in coagulation parameters compared to wild-type controls. Moribund *cbs* (−/−) mice exhibited severe liver injury and hepatic fibrosis while surviving *cbs* (−/−) mice although less severely affected, still exhibited a level of severe liver injury that is not found in the human disease. The hepatopathy observed in this model may offer an explanation for the failure of *cbs* (−/−) mice to respond to betaine or exhibit a hypercoagulative phenotype. We conclude that although this model provides useful data on the biochemical sequelae of classical homocystinuria, it does not successfully recapitulate a number of important features of the human disease and its use for studying mechanisms in homocystinuria should be treated with caution as the hepatopathy produces changes which could influence the results.

## Introduction

Cystathionine beta-synthase (EC 4.2.1.22, CBS) is a pyridoxal 5′-phosphate (PLP)-dependent heme protein that catalyzes the condensation of serine and homocysteine (Hcy) to form cystathionine as the first committed step in cysteine biosynthesis by transsulfuration. Cystathionine thus formed is subsequently converted to cysteine and α-ketobutyrate by the action of cystathionine γ-lyase (CGL) [1]. The transsulfuration pathway is limited to liver, kidney, CNS, small intestine and pancreas [2]. Inactivation of CBS results in classical homocystinuria (HCU), which is the most common inherited defect in sulfur amino acid metabolism and is characterized biochemically by severe increases in plasma and tissue levels of Hcy, methionine and *S*-adenosylhomocysteine (AdoHcy). Conversely, the plasma concentration of metabolites distal to the block, cystathionine and cysteine are decreased. The incidence of this disease appears to vary significantly among populations with more recent estimates of birth prevalence, i.e., calculated homozygosity or compound heterozygosity for pathogenic mutations ranging from between 1 in 6,400 and 20,500 [3–6].

The clinical manifestations of untreated HCU include mental retardation, thromboembolism and connective tissue defects including lens dislocation, osteoporosis and a range of characteristic skeletal abnormalities. Current treatment for pyridoxine non-responsive HCU in humans typically involves a combination of dietary supplementation with betaine and restricted dietary intake of the Hcy precursor methionine. Betaine lowers Hcy levels by serving as a methyl donor in the remethylation of Hcy to methionine catalyzed by betaine-homocysteine *S*-methyltransferase (BHMT) [1].

Analysis of the pathological mechanisms that underlie this disease is hampered by the unavailability of tissues from patients and ethical considerations concerning patient treatment. To date, the majority of published research on the pathophysiological mechanisms induced by HCU has been based upon a previously described CBS knockout mouse model [7]. These *cbs* (−/−) animals suffer from pronounced liver injury and typically die within 2–3 weeks of birth [7]. This neonatal semi-lethality is not mirrored in human patients and restricts the utility of the model. In this paper, we investigate if the utility of the *cbs* (−/−) mouse model can be improved by either treatment with the Hcy lowering agent betaine or the downstream metabolite cysteine. We report here that the birth incidence of the *cbs* (−/−) mouse is non-Mendelian and that neither cysteine nor betaine significantly improves the birth incidence of these mice. Betaine improved the survival of *cbs* (−/−) mice and restored fertility to female *cbs* (−/−) mice but without significantly lowering Hcy. Surviving *cbs* (−/−) mice failed to show any alteration in coagulation parameters compared to wild-type controls and exhibited severe liver injury, steatosis and fibrosis that was not significantly improved by either betaine or *N*-acetylcysteine treatment. We conclude that while *cbs* (−/−) mice exhibit a metabolic profile that is similar to the human disease, this model should be used with caution for studying mechanisms in HCU as the profound hepatopathy in this model produces changes which could influence results.

## Materials and methods

### Animal studies

A breeding pair of heterozygous *cbs* (+/−) mice was obtained from the Jackson laboratory and was subsequently maintained on standard chow (LabDiet NIH5K67, PMI nutrition international, Brentwood, MO). To minimize the potential influence of differences in genetic background, mice were crossbred to C57BL/6J mice (The Jackson Laboratory, Bar Harbor, Maine) for 7 generations. For rescue experiments, trimethylglycine (betaine) or the cysteine donor compound *N*-acetylcysteine (NAC) (Sigma-Aldrich) was dissolved in drinking water (20 g/l) and supplied *ad libitum* to breeding pairs of heterozygous *cbs* (+/−) mice prior to conception. This treatment was continued through birth and until final weaning. Survival curves and weights were recorded for all mice in all treatment and control groups up until weaning at 21 days. All experiments were approved by the University of Colorado Health Sciences center institutional animal care and use committee and were performed according to the NIH standards for animal care and use.

### Genotyping

Genomic DNA for genotyping was prepared from tail biopsies using the Mouse Tail Kit D-7000B from Puregene (Minneapolis, MN) according to the manufacturer's instructions. All PCR reactions were performed using Herculase polymerase (Stratagene) and a Stratagene Robocycler. DNA for genotyping was amplified in 2 different reactions.

Primer no. 614 5′-TCA GAA CCA AGA GCC AGC-3′ derived from intron 2 of the mouse *cbs* gene and primer no. 615 5′-CTT CCC CTT TTG ACC TCC-3′ from exon 3 were used to examine for the presence or absence of mouse *cbs* exon 3 which is not present in homozygous *cbs* (−/−) mice. This reaction was performed using 2 min at 92° followed by 30 cycles of 92 °C for 30 s, 61 °C for 30 s, 68 °C for 2 min and 30 s followed by 5 min at 68 °C. A second PCR reaction was performed using primer nos. 616 5′-GCC TCT GTC TGC TAA CCT A- 3′ and 392 5′-GAG GTC GAC GGT ATC GAT A-3′ to test for the presence or absence of the neo gene replacing exons 3 and 4 in the heterozygous and homozygous knockout mice. The amplification conditions for the detection of the neo gene were 92 °C for 2 min followed by 92 °C 30 s, 60 °C 30 s, 68 °C 60 s followed by 10 min at 68 °C. All *cbs* (−/−) mice had their genotype independently confirmed by amino acid profiling to determine total homocysteine (tHcy) and cystathionine levels (see below) in plasma samples acquired by non-lethal tail bleeding.

### Thiols and methionine cycle metabolite measurements

Determination of plasma levels of amino acids and AdoMet and AdoHcy was performed as described previously [8,9]. In mouse tissues, the levels of free aminothiols (i.e., non-protein bound aminothiols) were determined to prevent rapid enzyme-mediated turnover of aminothiols during sample preparation by immediate deproteination of tissue samples. Typically, 100-mg samples of frozen tissue (wet weight) were ground into powder in liquid nitrogen and were then homogenized in 450 μL of 0.4 N perchloric acid (PCA) containing 1 mM EDTA. Subsequently, the homogenate was centrifuged (7000 ×*g* 10 min) and 100 μL of supernatant was mixed with 80 μL of internal standard solution (*N*-(2-mercaptopropionyl)-glycine, 2 mg/l) and reduced with 25 μL of Tris (2-carboxyethyl) phosphine (TCEP) (120 mg in 1 mL of PBS) for 30 min at room temperature. After this reduction step, 15 μL of the mixture was mixed with 100 μL of derivatization solution (ammonium7-fluorobenzo-2-oxa-1, 3-diazole-4-sulfonate (SBD-F) in borate-EDTA buffer, 0.125 mol/l–4 mmol/l) and was then incubated for 30 min at 60 °C. A 10-μL aliquot of this derivatized sample was injected onto an RP C18 column (Prontosil C18-AQ, 250 × 4.0 mm, 3.0 μm, Bischoff Chromatography, Germany) and the fluorescence intensities of derivatized aminothiols were measured with excitation at 385 nm and emission at 515 nm.

### Histological examination of mouse tissues and assessment of hepatic function

Mice were sacrificed by decapitation and selected tissues were immersion-fixed overnight in 4% paraformaldehyde in PBS (pH 7.3). Paraffin-embedded sections were stained with hematoxylin and eosin to evaluate gross histopathological changes including steatosis and inflammation. Masson trichrome staining was performed to assess changes in collagen deposition and fibrosis. For ultrastructural studies, parallel samples of the liver were post-fixed in 1% phosphate-buffered (pH7.4) OsO4, dehydrated in ethanol and embedded in Epon-araldit with uranyl acetate and lead hydroxide and viewed on a Tesla 500 electron microscope (Tesla, Czech Republic).

Hepatic hydroxyproline content was determined as an index of fibrosis as described previously [10]. Liver injury was assessed by determining plasma levels of alanine aminotransferase (ALT) activity using an enzyme-coupled assay with lactic dehydrogenase (LDH) as described previously [11]. Liver lipid was extracted using the procedure of Bligh and Dyer [12], and after evaporation of the organic solvent, the triacylglycerol content of each sample was measured in duplicate using an enzymatic method (Sigma-Aldrich).

### Assessment of coagulation parameters

The coagulative phenotype of *cbs* (−/−) mice was assessed by determination of tail bleeding times using a previously reported method as a surrogate of hemostasis and thrombosis function [13]. Possible alterations in the extrinsic coagulation pathway were investigated using the prothrombin time (PT) assay as described previously [14]. Possible quantitative and qualitative abnormalities in the intrinsic and common pathways of coagulation were investigated by determining the activated partial thromboplastin time (aPTT) [14]. For these analyses, mice were anesthetized with pentobarbital (50 mg/kg intraperitoneally), and venous blood was collected via direct right atrial puncture. Plasma samples (20 μl) were diluted with 80 μl of water to a final volume of 100 μl and assayed in an electromechanical ST4 coagulation analyzer (Diagnostica Stago, Parsippany, NJ) according to the manufacturer's standard protocol.

### Statistical methods

Mendelian birth incidence of mouse genotypes in the presence and absence of treatment was examined using chi-square analysis. The Kaplan–Meier survival curves were constructed using Prophet 5.0. Differences in survival functions were tested by Mantel–Cox test using the same software. All tests were Bonferonni corrected where appropriate; significance level for tests was set at *p <* 0.05. For the tail bleeding time experiments, Kolmogorov–Smirnov tests indicated that the data were not normally distributed and subsequent statistical analysis of between group differences were performed using the non-parametric Kruskal–Wallis test. All other data are presented as means ± SD. Statistical analyses were performed by using the unpaired Student's *t* test. A *p* value of less than 0.05 was considered statistically significant. In the graphed data, *p < 0.05, **p < 0.01 and ***p < 0.001.

## Results

### Birth incidence of *cbs* (−/−) and *cbs* (+/−) mice is not Mendelian

**Fig. 1:**
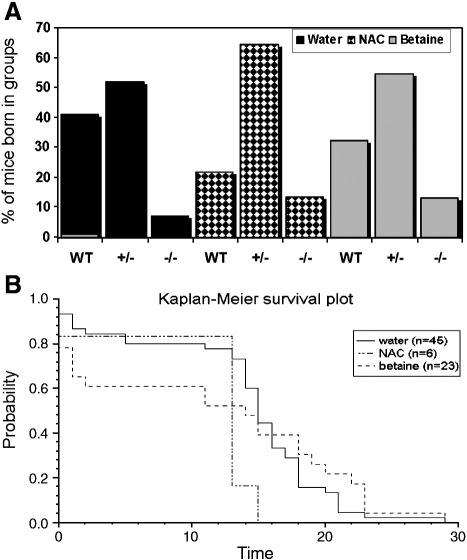
(A) The birth incidence of *cbs* null (−/−) mice in the presence and absence of betaine or NAC treatment is non-Mendelian indicating possible *in utero* selection. Genotyping of mice was performed as described in the Materials and methods section. Betaine and NAC treatment was administered for at least 1 week before conception and continued until weaning as described in the Materials and methods section. (B) Kaplan–Meier analysis of the influence of NAC and betaine upon survival of *cbs* (−/−) mice. The *x*-axis denotes survival time given in days *post partum*. Water v betaine, *p* = 0.02. Water v NAC, *p* = 0.001.

In the original report describing the generation of the *cbs* (−/−) *mouse* model, the birth incidence of *cbs* (+/−) and *cbs* (−/−) mice was reported to be Mendelian [7]. This conclusion was based on a sample size of 273 pups observed at weaning. In our analysis, we have analyzed the proportion of genotypes of a total of 605 mice born to untreated *cbs* (+/−) parents. Mice were checked twice a day and any dead mice were removed and genotyped. This analysis (Fig. 1A) found that the birth incidence of the *cbs* (−/−) genotype is unequivocally non-Mendelian as assessed by chi-square analysis (52 observed vs. 150 expected; *p* < 0.001). Similarly, the ratio of the *cbs* (+/−) to wild-type was significantly lower than the expected 2:1 ratio (329:223 vs. 301:150 expected *p* < 0.01). Both of these findings are strongly suggestive of *in utero* selection associated with both mild and severe CBS deficiency. These findings differ significantly from the previous analysis of this mouse model and a possible explanation for this discrepancy was suggested by our initial pilot experiments. We found that in our laboratory, the PCR conditions described previously were not reliable and that many mice that appeared to be *cbs* (−/−) by PCR analysis were found to be *cbs* (+/−) when bled and analyzed for tHcy. In order to avoid this possible error for the analysis presented here, we designed new primers and conditions for genotyping and confirmed genotype by tail bleeding and subsequent tHcy determination.

In terms of longevity, approximately 90% of untreated *cbs* (−/−) mice died within the early neonatal period. Because young mice cannot be reliably marked for identification during this time, the genetic identity of the *cbs* (−/−) mice was only determined after the animal had died. The low incidence of *cbs* (−/−) mice and their extremely short average life span combine to severely limit the utility of this model for the purpose of studying the pathophysiology of HCU.

### Betaine improves the survival but not the birth incidence of *cbs* (−/−) mice

Current treatment for pyridoxine non-responsive HCU in humans typically involves a combination of dietary supplementation with betaine and restricted dietary intake of the Hcy precursor methionine. In terms of survival and clinical outcome, betaine and dietary protein restriction have proven to significantly lower Hcy and improve clinical outcome in human patients [1].

To investigate if either betaine or the end product of the transsulfuration pathway cysteine could improve either the birth incidence and/or longevity of *cbs* (−/−) mice, the experiment described above was repeated using *cbs* (+/−) breeding pairs treated with either 2% (w/v) betaine or 2% (w/v) *N*-acetylcysteine (NAC) supplied *ad libitum* in drinking water.

The treatment of the parent mice with NAC and analysis of the genotype of their progeny (*n* = 62) indicated that this treatment served to improve the birth incidence of the *cbs* (+/−) mice to Mendelian levels with respect to wild-type (40:14 vs. 36: 18 expected) (Fig. 1A). The birth incidence of *cbs* (−/−) mice born was slightly improved compared to those observed without treatment, but despite this improvement, the birth incidence of *cbs* (−/−) was still a significant deviation from the predicted Mendelian incidence (6 mice instead of the 15.5 expected; *p* < 0.05), but notably, no *cbs* (−/−) mouse from this treatment group survived past 15 days.

Betaine treatment (*n* = 325) also restored the birth incidence of *cbs* (+/−) mice to the expected Mendelian levels with respect to wild-type (177:105 vs. 188:94 expected). The birth incidence of the *cbs* (−/−) phenotype was still distinctly non-Mendelian (23 vs. 81 expected *p* < 0.001). At the end of the trial, 6 of the 23 cbs (−/−) mice born to heterozygous *cbs* (+/−) parents on betaine survived for longer than 2 weeks (Fig. 1B). Kaplan–Meier survival analysis was used to compare the survival rates of cbs (−/−) mice as a consequence of betaine or NAC treatment (Fig. 1B). This analysis indicated that betaine significantly improved survival of *cbs* null mice (*p* = 0.02). A significant difference was observed between the water and NAC group (*p* = 0.001), but in this case, it appears the treatment acted to decrease survival.

### Betaine treatment allows *cbs* (−/−) mice to conceive and deliver pups but transsulfuration is required for lactation

One way that the birth incidence of *cbs* (−/−) mice could be increased would be to breed surviving *cbs* (−/−) mice with each other such that all progeny would be *cbs* (−/−). In the original report of this mouse model, *cbs* (−/−) female mice were reported as being infertile [7]. Subsequent work has indicated that male *cbs* (−/−) mice are fertile and can be used in mating experiments but confirmed that female *cbs* (−/−) mice are infertile. The ovaries of *cbs* (−/−) mice were reported as being free from gross abnormality and that their infertility was a consequence of uterine failure possibly due to the deleterious effects of elevated Hcy [15]. We investigated if female *cbs* (−/−) mice could conceive and deliver pups in the presence of betaine treatment. Breeding pairs of two male *cbs* (−/−) and four female *cbs* (−/−) mice were pre-treated with betaine in drinking water as described above for 1 week before being allowed to mate. Betaine treatment was continued throughout the resulting pregnancies. Under these conditions, we found that all of the female *cbs* (−/−) mice were able to conceive and progress to full-term delivery of small litters of between 2 and 4 pups. PCR genotyping confirmed that all of the pups born from these crosses were *cbs* (−/−) mice. The pups born to these mice all died within 1 day of birth, as the *cbs* (−/−) mothers were all unable to lactate. Female *cbs* (−/−) mice were observed trying to feed their pups but dissection of the dead pups showed no evidence of any gastric milk. This experiment was repeated where in addition to betaine; the drinking water was supplemented with 2% (w/v) NAC in drinking water. This treatment successfully restored lactation in the *cbs* (−/−) mice and gastric milk was observed in the stomachs of the resultant pups. Despite the restoration of lactation, pups born to *cbs* (−/−) mice all died within 2 days of birth indicating that their viability was reduced.

### Assessment of hepatopathy in surviving *cbs* (−/−) mice

**Fig. 2:**
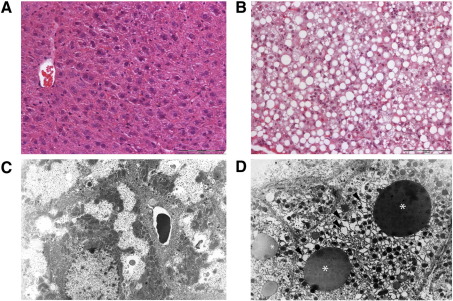
Histological and ultrastructural analysis of *cbs* (−/−) mouse liver. Representative sections showing hematoxylin and eosin mouse liver stains of (A) wild-type control and (B) surviving cbs (−/−) mouse livers. The surviving cbs (−/−) mouse livers show steatosis and occasional resorptive granuloma (scale bar denotes 200 micrometers). Panels C and D show representative electron micrographs of wild-type control and surviving cbs (−/−) mouse livers, respectively (8000× magnification). Surviving cbs (−/−) mouse livers exhibited lipid droplets (asterisks) with variable density and distended cisternae in the ER (black arrowheads).

Previous characterization of homozygous *cbs* (−/−) mice indicated that these mice suffered from hepatic steatosis [7]. Similarly, when we sacrificed betaine treated *cbs* (−/−) mice that had survived into adulthood, we found that the livers of these mice were severely enlarged and profoundly steatotic. Liver samples from these animals were processed for histological analysis by both optical (Figs. 2A and B) and electron microscopy (Figs. 2C and D) as described in the Materials and methods section. These liver samples showed a normal lobular structure, but hepatocytes were enlarged and markedly anisocytic. Nuclei were enlarged, binucleated and in a number of cases multinucleated with intranuclear pseudoinclusions and chromatin clumping. Many hepatocytes exhibited pronounced microvesicular to macrovesicular steatosis with the remaining parts of the cytoplasm appearing to be dense and solid. Multiple dispersed focal monocellular to oligocellular necroses were present with a resorptive inflammatory reaction. Electron microscopy of parallel samples of these tissues revealed that the cytoplasm of *cbs* (−/−) hepatocytes is dominated by an overall increase in the number of organelles and by variably distended cisternae of rough endoplasmic reticulum (ER) sometimes with fine intracisternal proteinaceous precipitates. In addition, numerous lipid droplets of varying density were observed in a number of hepatocytes. Collectively, our histological analysis showed that the surviving *cbs* (−/−) mice exhibit severe hepatopathy with marked signs of hepatocyte damage suggesting necrosis and secondary hyper-regeneration.

### Moribund *cbs* (−/−) mice exhibit severe liver injury and hepatic fibrosis

**Fig. 3:**
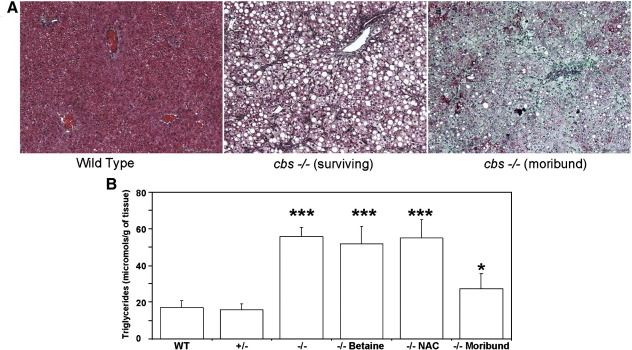
Severe hepatic steatosis and fibrosis in *cbs* (−/−) mice. Representative sections showing Masson trichrome staining of (A) wild-type control (left) surviving *cbs* (−/−) (center) and moribund *cbs* (−/−) (right) livers. Pictures shown are representative of 20 views taken from a minimum of 3 animals. Green stain denotes the presence of collagen. Scale bar denotes 200 micrometers. (B) Hepatic triglyceride content in wild-type (WT), *cbs* (+/−) and *cbs* (−/−) mice. *n* = 4 for each group. Statistical comparisons of *cbs* (−/−) mice were all made relative to hepatic triglyceride levels in wild-type mice. In this figure and all subsequent graphs presented here, *p < 0.05, **p < 0.01 and ***p < 0.001.

As approximately 90% of *cbs* (−/−) mice in our study exhibited neonatal lethality, the phenotype of the surviving *cbs* (−/−) mice is by definition atypical of the model as a whole. Therefore, the hepatic pathology of the surviving mice may differ significantly from that of the mice that die during the early neonatal period. In order to investigate why so many *cbs* (−/−) mice die during the early neonatal period, we sacrificed a number of litters prospectively and examined their livers without knowledge of genotype at the time of inspection. During this analysis, we examined a total of 9 mice with an average age of 12 days that were clearly failing to thrive and appeared to be moribund. Seven of these mice were subsequently confirmed as *cbs* (−/−) by PCR genotyping and by quantification of plasma tHcy levels in samples taken at sacrifice. These moribund *cbs* (−/−) mice showed stunted growth compared to littermates and in contrast to the surviving *cbs* null mice described above, their livers were not pink and enlarged but instead were white, shriveled and friable with a mottled appearance. Masson trichrome staining of formalin fixed sections of these livers showed clear evidence of neutrophil invasion and extensive fibrosis (Fig. 3A). Plasma samples derived from these mice were bright yellow suggesting the presence of bile pigments consistent with liver failure.

The transition from macrosteatosis to microsteatosis is a common observation in the etiology of end-stage liver disease as the liver transitions from the intermediate steatotic stage to the terminal fibrotic stage [16]. Visual inspection of the hematoxylin- and eosin-stained livers indicated that the degree of lipid accumulation in the moribund *cbs* (−/−) mice appeared to be decreased relative to the livers of the surviving *cbs* (−/−) mice. In an attempt to quantify this observation, we determined the hepatic triglyceride content of liver samples from moribund *cbs* (−/−) mice (*n* = 5) as described in the Materials and methods section and compared it to the values derived from the livers of surviving *cbs* (−/−) mice (*n* = 5). Additionally, to assess the possible therapeutic effects of betaine and NAC, we determined the lipid content of surviving *cbs* (−/−) mice that had been treated with these compounds for 1 month (*n* = 4 for each group). Wild-type and cbs (+/−) mouse livers (*n* = 10 for each) were used as controls. The result of this analysis (Fig. 3B) showed that the moribund *cbs* (−/−) mice have a significant decrease in hepatic triglyceride levels compared to surviving *cbs* (−/−) mice (*p* = 0.0003). Additionally, this analysis indicated that neither NAC nor betaine resulted in a significant reduction in hepatic triglycerides compared to surviving *cbs* (−/−) mice on water (*p* = 0.42 and 0.87 respectively). No significant increase in liver triglyceride level was observed in the heterozygous *cbs* (+/−) mice compared to controls (*p* = 0.43).

### Surviving *cbs* (−/−) mice exhibit mild fibrosis and liver injury

**Fig. 4:**
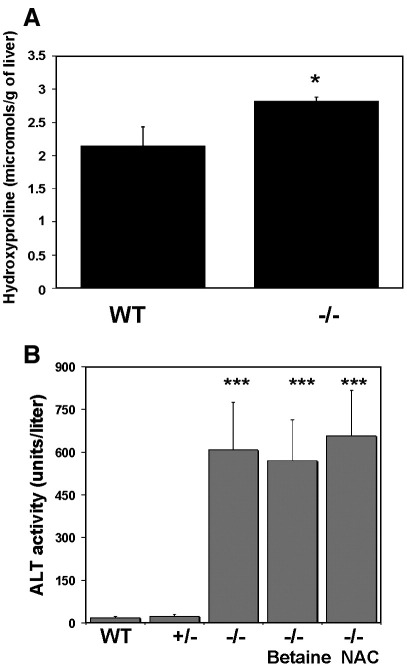
Fibrosis and liver injury in cbs (−/−) mice. (A) Hydroxyproline content in surviving *cbs* (−/−) mice (*n* = 3 for each group). (B) ALT levels in wild-type (WT), *cbs* (+/−) and *cbs* (−/−) mice. *n* = 5 for each group. Statistical comparisons of *cbs* (−/−) mice in this analysis were all made relative to ALT levels in wild-type mice (*p* < 0.0001 for all groups). No significant elevation of ALT levels was observed in *cbs* (+/−) mice.

Masson trichrome analysis suggested that the surviving *cbs* (−/−) mice exhibited some degree of fibrosis. We determined the hydroxyproline content of liver samples from surviving *cbs* (−/−) mice and compared it to the level found in wild-type control mice. Hydroxyproline is a major component of the protein collagen and as such can serve as an index of fibrosis in tissue samples. There was a statistically significant (*p* = 0.0154) increase in hepatic hydroxyproline content in the surviving *cbs* (−/−) mouse livers (Fig. 4A). The degree of hepatic injury incurred by surviving *cbs* (−/−) mice was assessed by determination of the plasma levels of ALT in wild-type, heterozygous *cbs* (+/−) and *cbs* (−/−) mice in the presence and absence of betaine or NAC (Fig. 4B). All of the *cbs* (−/−) groups showed a highly significant (*p* < 0.0001) increase in plasma ALT levels compared to the wild-type and heterozygous controls indicating relatively severe liver injury. The ALT levels did not vary significantly between the three *cbs* (−/−) groups indicating that neither betaine nor NAC significantly ameliorated liver injury in these mice.

### Assessment of coagulation parameters in *cbs* (−/−) mice

Thromboembolism is the major cause of morbidity in HCU [1]. In terms of increasing understanding of the mechanisms involved in HCU, it would be advantageous for any mouse model of this disease to recapitulate this aspect of the human phenotype. To assess the relevance of the *cbs* (−/−) model to the human disease, we used tail bleeding assays to assess thrombosis *in vivo* in wild-type and *cbs* (−/−) mice in the presence and absence of betaine treatment. The results of these experiments as assessed by a non-parametric Kruskal–Wallis test clearly demonstrated no significant deviation from the wild-type thrombosis phenotype in *cbs* (−/−) mice regardless of the presence or absence of betaine treatment (*p* = 0.37 and 0.72, respectively). Further investigation of coagulation parameters in *cbs* (−/−) mice was performed by testing the same mice for possible alterations in the extrinsic coagulation pathway by PT assay. Possible abnormalities in the intrinsic and common pathways of coagulation were investigated in the same mice by determining aPTT values. In agreement with the results for the tail bleeding analysis, the *cbs* (−/−) mice did not exhibit any statistically significant effect on either PT or aPTT. Collectively, our data indicates that the *cbs* (−/−) mouse model does not exhibit a hypercoagulative phenotype using these assays.

### Betaine does not significantly lower the plasma levels of Hcy in *cbs* (−/−) mice

**Fig. 5:**
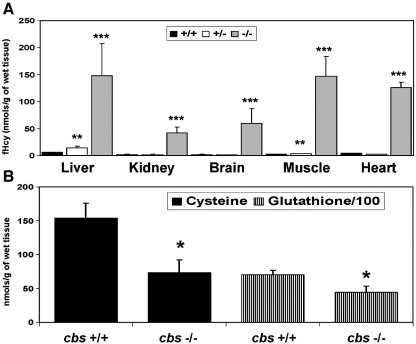
The metabolic consequences of CBS inactivation vary in different tissues. Tissue levels of A, fHcy and B, cysteine and glutathione in wild-type (WT), heterozygous (+/−) and *cbs* (−/−) mice. *n* = 4 for each group. Statistical comparisons of the values observed in heterozygous and *cbs* (−/−) mice in this analysis were all made relevant to tHcy, cysteine and glutathione levels in wild-type mice.

In an effort to elucidate the reason for the failure of the *cbs* (−/−) mouse model to exhibit altered hemostasis or the failure of betaine to influence the birth incidence of the null genotype, we performed an analysis of metabolites relevant to the methionine cycle in tissues and plasma of wild-type, *cbs* (+/−) and *cbs* (−/−) mice. For the tissue analysis, we determined the levels of non-protein bound Hcy, cysteine and glutathione in liver, kidney, brain, heart and calf muscle as described in the Materials and methods section. We observed (Fig. 5A) significant increases in the free homocysteine (fHcy) content in all of the tissues assayed from the *cbs* (−/−) mice compared to normal controls. The scale of the increase in fHcy varied significantly between the different tissues assayed in the *cbs* (−/−) mice. The liver, brain and kidney exhibited a 24-, 30- and 19-fold increase in fHcy levels, respectively. It is interesting to note that tissues that do not normally express the transsulfuration pathway such as heart and muscle exhibited very high increases in fHcy content in the *cbs* (−/−) mice (31- and 63-fold, respectively). With regard to *cbs* (+/−) mice, there was a statistically significant increase in the fHcy content of the liver compared to wild-type controls (*p* = 0.02), but there was no significant increase in any of the other tissues assayed. We observed statistically significant depletion of cysteine and glutathione in the livers of the *cbs* (−/−) (*p* = 0.007 and 0.003, respectively) compared to WT mice (Fig. 5B). There was no statistically significant difference in the cysteine or glutathione levels in any of the other tissues investigated.

In our plasma analysis, we determined the relative levels of tHcy, methionine, cysteine, cystathionine, alpha-ketobutyrate, serine, glycine, dimethylglycine (DMG), methylglycine (MG), AdoMet and AdoHcy in wild-type, *cbs* (+/−) and *cbs* (−/−) mice (Table 1). The *cbs* (−/−) mice exhibited significant increases in plasma tHcy, methionine, AdoMet and AdoHcy levels (*p* < 0.0001 for all four metabolites) compared to wild-type controls. In the same animals, cystathionine levels were effectively reduced to zero and cysteine was also reduced by approximately 50% (*p* < 0.0001).

**Fig. 6:**
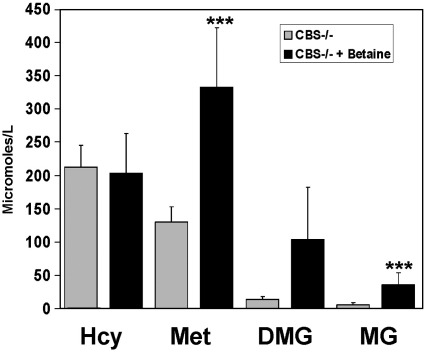
Biochemical effects of betaine treatment in *cbs* (−/−) mice. Plasma levels of tHcy, methionine (Met), dimethylglycine (DMG) and methylglycine (MG) in *cbs* (−/−) mice in the presence and absence of betaine (*n* = 7 for each group).

In our search for an explanation for the failure of betaine to rescues the semi-lethal phenotype of the *cbs* (−/−) mice, we performed an analysis of the plasma methionine cycle metabolites of *cbs* (−/−) mice in the presence and absence of 4 weeks of betaine treatment. We observed that betaine treatment resulted in a significant increase in plasma methionine (*p* = 0.0027), DMG (*p* = 0.02) and MG (*p* < 0.0001) levels (Fig. 6), indicating that the compound has been absorbed from the drinking water and that some remethylation of Hcy has taken place. However, our analysis revealed that betaine treatment did not significantly reduce the plasma level of tHcy in *cbs* (−/−) mice (*p* = 0.754).

## Discussion

Conventional treatment for pyridoxine non-responsive HCU has not advanced significantly since the introduction of methionine restricted diets and betaine therapy over 30 years ago. In order to improve treatment, there is a need to increase our knowledge of the pathogenic mechanisms that underlie the disease. It would therefore be useful to have a reliable animal model that faithfully recapitulates the major clinical sequelae without incurring any additional problems that have the potential to interfere or confound mechanistic studies. In terms of the biochemical perturbations, the untreated *cbs* (−/−) mice appear to be a reasonable model of the human disease. (Table 1 and Fig. 6). Inactivation of the mouse *cbs* gene results in an approximate 60-fold increase in plasma tHcy levels compared to wild-type controls while cystathionine levels were reduced to a level approaching the limits of accurate detection, indicating that diet is not a significant source of this compound for these mice. Plasma cysteine levels were also reduced by approximately 50% in *cbs* (−/−) mice representing a similar scale of reduction to that observed in human patients [17]. However, some differences were apparent; for instance, the plasma level of methionine was only increased approximately 2-fold in *cbs* (−/−) mice representing a less severe level of hypermethioninemia than that which is typically observed in human subjects with HCU [17]. Conversely, plasma AdoMet was elevated approximately 3-fold while plasma AdoHcy was increased some 25-fold with an average value of 4216 nM in the *cbs* (−/−) mice. This latter figure is approximately 4-fold higher than that typically observed in human subjects with HCU using identical methodology [17]. These perturbations result in an AdoMet/AdoHcy ratio of 0.35, representing a considerable change from that, observed for wild-type controls (2.8) and MKO *cbs* (+/−) mice (3.0), respectively. Interestingly, previous work in yeast has suggested that such a profound change in the AdoMet/AdoHcy ratio is inconsistent with cell viability and growth [18] and might suggest a possible contributory mechanism for the observed decreased viability of the *cbs* (−/−) mice.

Our analysis of tissue thiols found that *cbs* (−/−) mouse tissues exhibit significant differences in tHcy content compared to controls and that the scale of changes varies considerably in different tissues within *cbs* (−/−) mice. In our analysis, the liver and brain showed severe increases in tHcy levels while the increase in kidney tissue was relatively mild. Interestingly, tissues such as muscle, which do not express CBS and CGL, also experienced profound increases in Hcy levels. This observation is consistent with the fact that many of the clinical perturbances induced by inactivation of CBS manifest in tissues such as the skeleton and endothelium where transsulfuration is absent. In our analysis of tissue thiols, it was only in the *cbs* (−/−) mouse livers where we observed statistically significant depletion of cysteine and glutathione (*p* = 0.007 and 0.003 respectively) relative to wild-type controls. It is conceivable that the role of the liver in synthesizing and exporting glutathione into the bloodstream could act to exacerbate depletion of this compound as a consequence of the block in transsulfuration.

The liver pathology of the *cbs* (−/−) mice is analogous to that observed in a number of other hepatic diseases involving oxidative stress with a natural progression from hepatic steatosis (as an intermediate and reversible stage) to fibrosis–cirrhosis [16,19]. Hepatic steatosis has been reported in HCU in humans before [20] but the degree of liver injury described is likely to be very different to that found in this *cbs* (−/−) mouse model. Mild hepatic steatosis is an essentially benign condition that is relatively common in the general population [19]. To our knowledge, there has never been any report of elevated liver enzymes indicating liver injury in patients with HCU nor any documented case of hepatic fibrosis and/or liver failure in this disease. In this context, it is interesting to note that a recent report described the presence of pulmonary fibrosis in this *cbs* (−/−) model [21] indicating that fibrosis is not limited to the liver. Again, to date, there has been no report of any pulmonary impairment in patients with HCU adding to the speculation that this represents an artifact of the mouse model rather than a true reflection of the pathophysiology of the human disease. Similarly, a previous report has indicated that inactivation of CBS in this model acts to cause dysregulation of lipid metabolism [22]. Our findings presented in this paper raise the question as to how much of the observed dysregulation of lipid synthesis is a consequence of the profound steatosis that is observed in this mouse and brings into question the relevance of these previous findings to the human disease.

The possible presence of fibrosis in non-hepatic tissues in the *cbs* (−/−) mouse model presents a particular problem for any possible study of fibrillinopathy in HCU. Because of phenotypic similarities with the connective tissue disorders frequently observed in Marfan syndrome, it is likely that HCU involves impairment of fibrillin-1 function. In addition to being a component of extracellular matrix, a growing body of evidence indicates that fibrillin-1 serves to regulate transforming growth factor beta (TGF-beta) activation and signaling by sequestering and serving as a reservoir for this regulatory cytokine [23]. TGF-beta is the most potent and ubiquitous pro-fibrogenic cytokine and its expression is increased in all the fibrotic diseases and experimentally induced fibrosis models studied to date [24]. Given the level of fibrosis observed in the *cbs* (−/−) mice, it is highly likely that TGF-beta expression and activation is altered compared to control mice and serves as an example of how the fibrotic phenotype in the *cbs* (−/−) mouse could interfere with its use to study the pathogenic mechanisms of the disease as it occurs in humans.

Our study found that betaine treatment conferred some beneficial effects upon *cbs* (−/−) mice but it did so without significantly altering the tHcy level of the model. Our data implicate hepatopathy as the primary reason for the semi-lethal phenotype of *cbs* (−/−) mice and betaine has been shown to have hepatoprotective effects in both nonalcoholic steatohepatitis and alcoholic liver disease independent of its role in lowering Hcy [25,26]. It is thus conceivable that it is solely the hepatoprotective properties of betaine that are responsible for the improved survival of *cbs* (−/−) mice. Similarly, the ability of betaine to restore the fertility of female *cbs* (−/−) mice is probably also independent of Hcy. In this context, future studies should be directed towards examining the ovaries and endometrium of *cbs* (−/−) mice for evidence of fibrosis and if present, investigating if it can be reversed by betaine treatment. The observation that female *cbs* (−/−) animals are unable to lactate unless treated with NAC is consistent with previously published work by Zaragoza and co-workers [27]. This group has previously reported that lactation in rats is abolished if transsulfuration is blocked by treatment with CGL inhibitor compound propargylglycine. The decreased birth incidence of heterozygous animals cannot be explained by hepatic injury as their livers appeared to be normal. One possibility is the decreased incidence of heterozygous mice is due to early embryonic loss as a consequence of the elevated Hcy. Previous work using an avian embryo model system has reported dose-dependent teratogenic effects of exogenously added Hcy resulting in subsequent dysmorphogenesis resulting in defects in heart septation and/or neural tube closure. Supplementation with folic acid was found to mitigate the rise in Hcy and prevented the teratogenic effects [28]. Further work is needed to investigate if the observed decreased birth incidence of *cbs* (+/−) mice is due to embryonic loss induced by teratogenic effects of elevated Hcy.

Betaine has proven to be an effective treatment for lowering Hcy levels in human patients. Betaine lowers Hcy in mammals by serving as a methyl donor in the hepatic remethylation of Hcy catalyzed by BHMT which is expressed primarily in the liver and to a lesser extent the kidney [29]. Betaine does appear to be absorbed from the drinking water in our experiments and it appears to be functioning in the remethylation of Hcy as evidenced by the increased plasma levels of methionine and the betaine metabolites DMG and MG concomitant with this treatment (Fig. 6). However, it would appear that the level of BHMT activity is insufficient to significantly lower plasma tHcy levels in the *cbs* (−/−) mice. A possible explanation for the failure of betaine to lower Hcy levels in the *cbs* (−/−) mice comes from previous work which has shown that BHMT expression goes down to as low as 10% of normal levels in fibrotic/cirrhotic liver in humans [30]. Significantly decreased BHMT expression as a consequence of the observed hepatopathy might explain why this treatment fails to significantly lower Hcy in the *cbs* (−/−) mice.

To our knowledge, no investigation of the coagulative phenotype of the *cbs* (−/−) mouse model has been published to date. This is likely to be due to the difficulty in generating sufficient numbers of *cbs* (−/−) mice to perform the relevant experiments. The liver plays a central role in hemostasis and coagulopathy is frequently observed in acute liver disease. It is conceivable that the pro-coagulant effects of elevated Hcy are being masked in the *cbs* (−/−) mice as a consequence of coagulation abnormalities due to the severe hepatopathy. This possibility is consistent with a recent report that examined the effect of genetic background in *cbs* (−/−) mice. In this work, it was reported that those genetic backgrounds that ameliorated the hepatopathy were also found to exhibit endothelial dysfunction [31].

Taken collectively, the failure of the *cbs* (−/−) mice to respond to betaine or exert a hypercoagulative phenotype reported in this paper serve as examples of how severe hepatopathy may be acting to impair the ability of these mice to serve as an effective model of the human disease. In the future, it may prove that this model has greater utility for testing treatment strategies for end-stage fibrotic liver disease rather than HCU. We conclude that problems with fecundity, viability and the possible confounding effects of growth retardation, fibrosis and liver failure severely limit the utility and relevance of this model for studying the human disease. Previous investigations of pathogenic mechanisms using this model should be considered with caution as the hepatopathy incurred could influence results.

## Figures and Tables

**Table 1:** Plasma concentrations of metabolites relevant to transsulfuration in wild-type, heterozygous and *cbs* null mice.

Genotype	Regimen	tHcy	Cystat	Met	tCys	Ser	DMG	Gly	ABUT	MG	AdoMet	AdoHcy	AdoMet/AdoHcy
		μM	nM	μM	μM	μM	μM	μM	μM	μM	nM	nM	
**+/+**	Water	3.48 (0.46)	2479 (293)	62.9 (5.46)	214.0 (11.1)	179.0 (8.0)	10.0 (1.4)	356.0 (24.7)	3.98 (0.50)	2.39 (0.29)	707 (254)	251 (165)	2.80
**+/+**	NAC	4.50 (0.28)	2943 (190)	95.0 (20.7)	348.0 (66.0)	225.0 (26.8)	17.4 (0.8)	568.0 (58.9)	6.63 (0.80)	3.61 (0.18)	N.D.	N.D.	N.D.
**+/+**	Betaine	2.74 (0.24)	2444 (514)	66.0 (5.9)	183.0 (10.7)	206.0 (12.3)	102.0 (14.4)	365.0 (38.3)	4.20 (0.37)	13.6 (1.16)	469 (122)	167 (37)	2.80
**+**/−	Water	8.30 (0.77)	4968 (824)	87.0 (5.2)	227.0 (9.1)	177.0 (7.4)	10.7 (0.7)	396.0 (26.8)	4.50 (0.20)	2.70 (0.24)	1331 (138)	539 (208)	2.46
**+**/−	NAC	6.98 (1.1)	2384 (148)	63.0 (16.0)	222.0 (51.2)	168.0 (21.0)	15.0 (0.8)	468.0 (47.0)	6.00 (0.80)	3.53 (0.39)	N.D.	N.D.	N.D.
**+**/−	Betaine	8.13 (0.73)	4693 (723)	122.0 (15.0)	231.0 (8.3)	257.0 (15.7)	82.7 (8.0)	386.0 (28.0)	5.80 (0.90)	26.4 (2.27)	757 (135)	251 (52)	3.00
−/−	Water	212.6 (32.9)	212.2 (115.0)^⁎^	130.0 (23.2)	117.8 (8.8)	172.20 (24.3)	13.8 (4.5)	272.0 (92.0)	4.00 (0.19)	6.00 (2.96)	1498 (86)	4216 (586)	0.355
CBSDH patients^a^		155–471	0–79	353–1891	40–140	N.D.	3.5–6.4	N.D.	N.D.	3.6–15.1	888–2030	147–1700	N.D.
Normal range^b^		5.4–13.9	50–342	13–45	200–361	N.D.	1.4–5.3	N.D.	N.D.	0.6–2.7	59–120	9–21	N.D.

Values shown represent the average value derived from between 7 and 20 animals with the standard deviation given in parentheses. ^⁎^ Values for cystathionine in cbs null mice are below the limits of accurate detection and represent essentially background readings. ^a^ Determined from 7 untreated CBSDH patients on a normal diet [17]. ^b^ Determined for 60 normal subjects aged 18–65 years [17]. tHcy, total homocysteine; Cystat, cystathionine; Met, methionine; tCys, total cysteine; Ser, serine; DMG, dimethylglycine; Gly, glycine; ABUT, alpha aminobutyrate; MG, methylglycine; AdoMet, *S*-adenosylmethionine; AdoHcy, *S*-adenosylhomocysteine; N.D., not determined.
